# Characterization of the complete mitochondrial genome of a barklouse, *Lepinotus* sp. (Psocodea: Trogiomorpha: Trogiidae)

**DOI:** 10.1080/23802359.2021.1930218

**Published:** 2021-05-23

**Authors:** Dan-Dan Wei, Yan-Qing Tu, Peng-Yu Guo, Jin-Jun Wang

**Affiliations:** a Key Laboratory of Entomology and Pest Control Engineering, College of Plant Protection, Southwest University, Chongqing, P. R. China; b Academy of Agricultural Sciences, Southwest University, Chongqing, China

**Keywords:** Psocids, barklice, mitochondrial genomes, tandem repeat, Phylogenetic analysis

## Abstract

Barklice in the genus *Lepinotus* (Psocoptera: Trogiidae) are small, soft-bodied stored-product pests that are difficult to control. We sequenced and annotated the mitochondrial (mt) genome of *Lepinotus* sp. The mt genome of *Lepinotus* sp. is 16,299 bp in size with 74.4% A + T content. The gene order was highly conserved in some of the Trogimorpha barklice. Two types of tandem repeat units were identified in CR of *Lepinotus* sp. The phylogenetic analysis showed that Trogiidae species was the sister group to Lepidopsocidae barklice, and the suborder Troctomorpha was polyphyletic.

Psocoptera (booklice and barklice, also named psocids) contains more than 5000 described species, including three suborders (Trogiomorpha, Psocomorpha and Troctomorpha) (Liu et al. 2017). The barklice from genus *Lepinotus* Heyden, 1850 (Psocoptera: Trogiidae) are stored-product pests worldwide (Arif et al. 2012). *Lepinotus* has more than 12 described species (Liang et al. 2012). To date, 13 complete or nearly complete mitochondrial (mt) genomes of barklice species have been reported (Shao et al. 2003; Li et al. 2013; Liu et al. 2017; Yoshizawa et al. 2018), and only one incomplete mt genome has been sequenced for *Lepinotus* species, i.e. *Lepinotus reticulatus* (Feng et al. 2018). To get further information about mt genomes of barklice species, we sequenced and analyzed the complete mt genome of a barklouse, *Lepinotus* sp.

*Lepinotus* sp. was collected from Chizhou city, Anhui Province, China in 2014 (30^°^66′N, 117^°^49′E). Voucher specimens were deposited in the Entomological Museum (Accession Number: SWU Ps-02-01-01) of the college of Plant Protection, Southwest University. Total genomic DNA was extracted using TIANamp Genomic DNA Kit (Tiangen Biotech, Beijing, China) and stored at −20 °C until future use. Fragments of *cox1*, *cytb*, *nad5* and *16S* were amplified by PCR using conserved insect primers (Simon et al. 2006), and *nad2* was derived from transcriptome data of this barklouse. Five overlapping fragments (*cox1-nad5*, *nad5-cytb*, *cytb-16S*, *16S-nad2* and *nad2-cox1*) were amplified by long PCR using the specific primers, which were designed from above-mentioned gene sequences. These fragments were assembled into contigs with SeqMan (DNAStar) and proofed manually. All products were sequenced by the Beijing Genomics Institute (BGI) at Chongqing, China.

The complete mt genome of *Lepinotus* sp. is a closed-circular molecule of 16,299 bp in size (GenBank accession number: MW735944), containing 13 protein-coding genes (PCGs), 22 tRNA genes, 2 rRNA genes and a control region (CR). *Lepinotus* sp. has the same gene arrangements and coding strand as in some published Trogimorpha barklice species, e.g. lepidopsocid sp., *L. reticulatus* and *Dorypteryx domestica* (Shao et al. 2003; Feng et al. 2018; Yoshizawa et al. 2018). The A + T content of complete mt genome of *Lepinotus* sp. is 74.4% (A = 38.8%, T = 35.9%, C = 17.9 and G = 7.4%), which is similar to other reported psocids (ranging from 68.6% to 79.8%). All PCGs of *Lepinotus* sp. initiated with ATD (six ATT, five ATG and two ATA) as the start codons, and mostly terminated with the stop codon TAA/TAG, except for *nad5* and *cytb* (end with the incomplete stop codon T-). The CR (1580 bp) of *Lepinotus* sp. is located between *rrnS* and *trnQ*, with an A + T content of 80.0%. Two types of tandem repeat units (TR1 and TR2) were identified in CR of *Lepinotus* sp., and the repeat units of TR1 and TR2 were 122 bp (seven times) and 21 bp (six times) in length, respectively. Interestingly, most of the barklice possess tandem repeats in their control region, and these repeat units are highly similar among the species from the same genus, e.g. *Lepinotus* sp. and *L. reticulatus*.

The phylogenetic analysis was performed based on the nucleotide sequences of 11 PCGs (except *nad6* and *nad4L*) and two rRNA genes (in a total length of 11,381 bp) from 20 psocids species. The mt genome sequence of *Aphis citricidus* was used as an outgroup (Wei et al. 2019). A GTR + I + G substitution model for the concatenated dataset was used as the best-fit model, which was determined by jModelTest 2.1.4 (Posada 2008). The phylogenetic tree was constructed using the maximum-likelihood (ML) method, which was performed using IQ-TREE web server with 1000 bootstrap replicates (Trifinopoulos et al. 2016). The ML tree (Figure 1) consistently supported those of previous mitochondrial phylogenetic studies (Feng et al. 2018; Yoshizawa et al. 2018). The ML tree showed that each family was recovered as a monophyletic group, and booklice (Liposcelidae) and barklice formed well-supported mono clades, respectively. The suborder Troctomorpha was polyphyletic because the family Amphientomidae was positioned in the suborder Psocomorpha.

**Figure 1.**
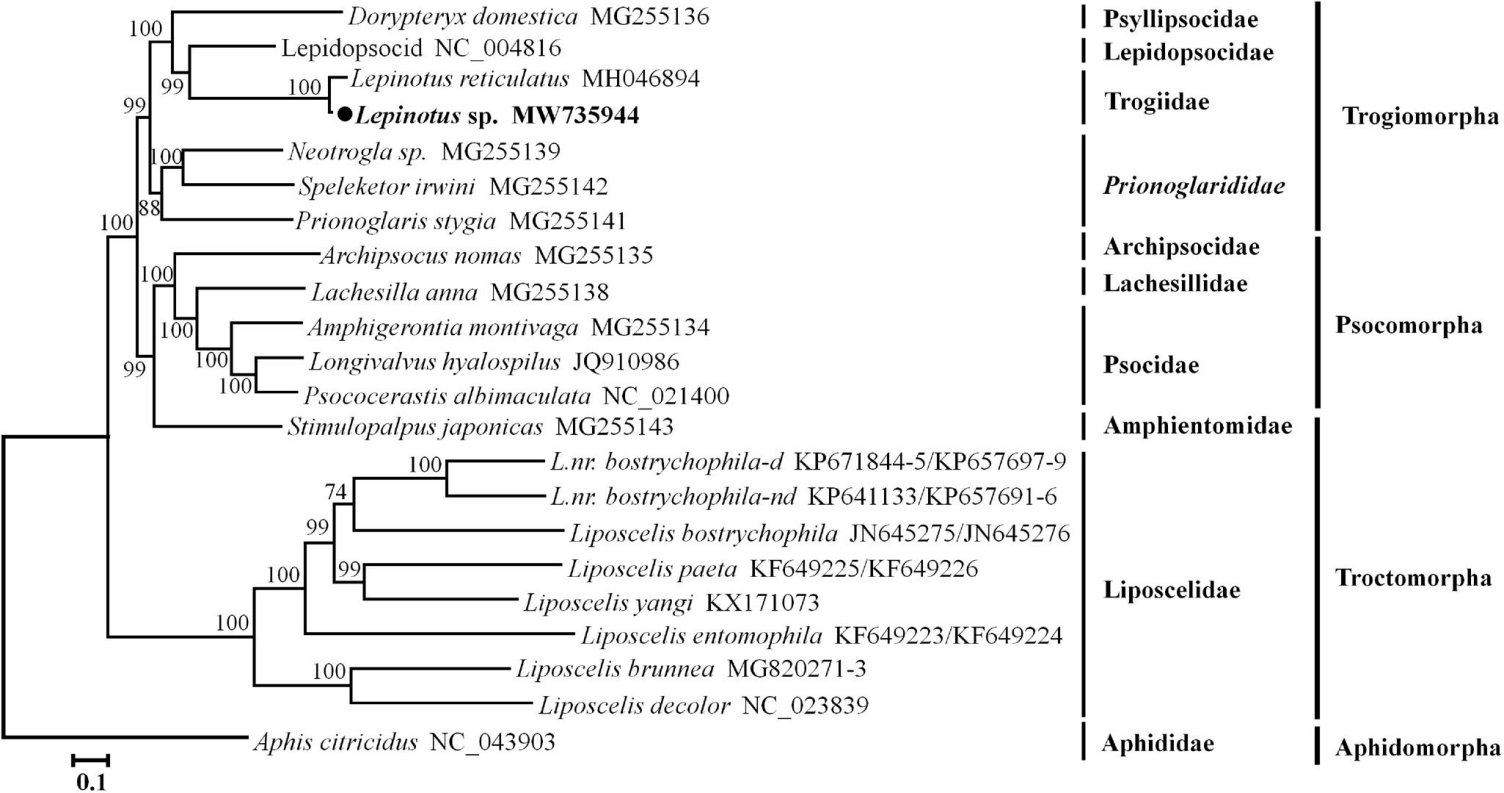
Phylogenetic relationships of psocids species inferred from nucleotide sequences of 11 PCGs and two rRNA genes of mitochondrial genomes based on the maximum-likelihood (ML) analysis. Numbers on branches are bootstrap support values. The accession numbers of each species are shown after the scientific name.

## Data Availability

The data that support the findings of this study are openly available in GenBank of NCBI (https://www.ncbi.nlm.nih.gov) with an accession number of MW735944.
